# Lameness Detection in Dairy Cows: Part 2. Use of Sensors to Automatically Register Changes in Locomotion or Behavior

**DOI:** 10.3390/ani5030388

**Published:** 2015-08-28

**Authors:** Annelies Van Nuffel, Ingrid Zwertvaegher, Stephanie Van Weyenberg, Matti Pastell, Vivi M. Thorup, Claudia Bahr, Bart Sonck, Wouter Saeys

**Affiliations:** 1 Technology and Food Science Unit, Precision Livestock Farming, Institute for Agricultural and Fisheries Research (ILVO), Burgemeester van Gansberghelaan 115 bus 1, 9820 Merelbeke, Belgium; E-Mails: ingrid.zwertvaegher@ilvo.vlaanderen.be (I.Z); stephanie.vanweyenberg@ilvo.vlaanderen.be (S.V.W.); 2 Natural Resources Institute Finland (Luke), Green Technology, Koetilantie 5, 00790 Helsinki, Finland; E-Mail: matti.pastell@luke.fi; 3 INRA, UMR 791 Systemic Modelling of Ruminant Nutrition, 16 rue Claude Bernard, 75231 Paris cedex 05, France; 4 AgroParisTech, UMR 791 Systemic Modelling of Ruminant Nutrition, 16 rue Claude Bernard, 75231 Paris cedex 05, France; E-Mail: vivi.thorup@agroparistech.fr; 5 Division Measure, Model and Manage BioResponses, Department of Biosystems, Katholieke Universiteit Leuven, Kasteelpark Arenberg 30 bus 2456, 3001 Heverlee, Belgium; E-Mail: Claudia.bahr@biw.kuleuven.be; 6 Animal Sciences Unit, Institute for Agricultural and Fisheries Research (ILVO), Scheldeweg 68, 9090 Melle, Belgium; E-Mail: bart.sonck@ilvo.vlaanderen.be; 7 Department of Biosystems Engineering, Faculty of Bioscience Engineering, Ghent University, Coupure links 653, 9000 Gent, Belgium; 8 Division Mechatronics, Biostatistics and Sensors (MeBioS), Department of Biosystems, Katholieke Universiteit Leuven, Kasteelpark Arenberg 30 bus 2456, 3001 Heverlee, Belgium; E-Mail: wouter.saeys@biw.kuleuven.be

**Keywords:** Lameness, early detection, on-farm, dairy cattle

## Abstract

**Simple Summary:**

As lame cows produce less milk and tendto have other health problems, finding and treating lame cows is very importantfor farmers. Sensors that measure behaviors associated with lameness in cowscan help by alerting the farmer of those cows in need of treatment. This reviewgives an overview of sensors for automated lameness detection and discussessome practical considerations for investigating and applying such systems inpractice.

**Abstract:**

Despite the research on opportunities toautomatically measure lameness in cattle, lameness detection systems are notwidely available commercially and are only used on a few dairy farms. However, farmers need to be aware of the lame cows in their herds in order treat themproperly and in a timely fashion. Many papers have focused on the automatedmeasurement of gait or behavioral cow characteristics related to lameness. Inorder for such automated measurements to be used in a detection system, algorithms to distinguish between non-lame and mildly or severely lame cowsneed to be developed and validated. Few studies have reached this latter stageof the development process. Also, comparison between the different approachesis impeded by the wide range of practical settings used to measure the gait or behavioralcharacteristic (e.g., measurements during normal farming routine or duringexperiments; cows guided or walking at their own speed) and by the differentdefinitions of lame cows. In the majority of the publications, mildly lame cowsare included in the non-lame cow group, which limits the possibility of alsodetecting early lameness cases. In this review, studies that used sensortechnology to measure changes in gait or behavior of cows related to lamenessare discussed together with practical considerations when conducting lamenessresearch. In addition, other prerequisites for any lameness detection system onfarms (e.g., need for early detection, real-time measurements) are discussed.

## 1. Introduction

To properly tackle the lameness problem, farmers need to be aware of the number of lame cows in their herd and the severity of their lameness. The commonly accepted methodologies to quantify lameness rely on identifying changes in the gait and posture of the cows and are discussed in the first part of this review [1]. In practice, this is done using subjective methods such as visual observations leading to locomotion scores by the farmer, an employee, a veterinarian or an agricultural consultant. Subjective locomotion scoring is quick to apply, inexpensive and easy to perform. However, it is time consuming when used to score the whole herd, and locomotion scoring is also subjective and frequently results in discrepancies between and within observers [2,3]. Moreover, most farmers underestimate the prevalence of lameness in their herd by a factor of four or more [4].

Since the 1980s, various sensors and technologies have been investigated for their potential to measure health indicators from individual cows [5]. Automated systems fit the trend of increasing herd sizes and less time for farmers to monitor the herds, let alone the individual cows. In addition, an automated lameness detection system might even be more sensitive than traditional observational methods for lameness detection [2].

Automatic measurement systems could support the dairy farmer and tackle the problem of the visual scoring systems, resulting in more objective measurements. However, the raw data collected from such sensor-based techniques still have to be translated into understandable gait variables that are functionally relevant for cattle gait (and therefore related to gait attributes used in locomotion scoring systems). This review summarizes several approaches to automatically measure lameness and related characteristics in dairy cows. The wide range of practical settings during the experiments, together with the definitions used to distinguish non-lame from lame cows, impede comparison between the different automated approaches. The factors obstructing such comparison and the need for specific characteristics (real-time/automated/continuous measurements, level of early detection, set-up size) of an automated lameness detection system are discussed.

## 2. Automatic Gait and Behavior Measurements and Lameness Detection

Several techniques have been proposed for automatic gait analysis, such as force platforms, electromyography, accelerometers and image-based technologies. In their review, Rutten *et al.* [6] divided the scientific publications into four categories: (I) techniques that measure something about the cow (e.g., activity); (II) interpretations that summarize changes in the sensor data (e.g., decrease in activity) to produce information about the cow’s status (e.g., lameness); (III) integration of information where sensor information is supplemented with other information (e.g., economic information) to produce advice (e.g., whether to treat a cow or not); and (IV) the farmer making a decision or the sensor system making the decision autonomously (e.g., the trimmer or veterinarian is contacted).

In 2013, 38 publications on lameness were described in the review by Rutten *et al.* [6], but only half of them included data interpretation, of which only one out of five had validated their algorithm, often without reporting the sensitivity and specificity. The main reason for this low fraction of validated algorithms is that most studies still focused on category I: gaining information on cow characteristics related to lameness using sensor technology. In the next section, different measuring techniques used to gain information on specific lameness-related characteristics in cows are summarized based on the used sensor technology. Only those peer reviewed papers that compared the sensor data with locomotion scoring as reference were considered. Other references such as lesion scoring were rarely incorporated, as this review focuses on the detection of lameness in general and not on the detection of hoof lesions.

### 2.1. Load Cells

#### 2.1.1. Measurement of Walking Cows

Rajkondawar *et al.* [7] developed a 1D ground reaction force system that measured ground reaction forces (GRF) when the cows walked over 2 parallel force platforms 1.98 m long and calculated 7 variables on the left and right hind legs of the cow. Both sides of the platform had 4 load cells under the floor plate. With this system the peak GRF was measured for 11 non-lame and 12 lame cows [8]. In 2006, more variables were added to the list for distinguishing lame from non-lame cows: peak GRF, average GRF, stance time, impulse (*i.e.*, integral of GRF over time), and area under the Fourier transformed curve of GRF [9].

Based on these publications, the automatic lameness detection system StepMetrix^®^ (BouMatic, Madison, WI, USA) was introduced into the market in 2008. The model in this system used 5 limb movement variables and calculated the probability of one of the hind limbs being lame (SMX score). The StepMetrix was tested in a field trial by Bicalho *et al.* [10]. They reported a high specificity (93.8%), but a low sensitivity (22.2%) using the commercial settings of the StepMetrix^®^. Hence, lame cows were still being misclassified by the model. Liu *et al.* [11] improved the specificity and to a lesser degree the sensitivity up to 43% by using transformed limb movement variables. However, in another study by Liu *et al.* [12] the sensitivity of this lameness detection system was still limited (51.9%). The authors ascribed these poor results to the failure of transferring load off the limb where the cow showed higher pain reactions or where locomotion abnormalities or lesions were scored.

Forces exerted by the hooves on the ground can be measured using force plates—usually installed in the ground—that give information in one or three dimensions (vertical, forward/backward and sidewards horizontal forces, Fz, Fy and Fx respectively). Useful information of the ground reaction force can only be collected when no more than one limb at a time is on the force plate [13]. In cattle, using a force plate only 0.52 m long, approximately 30 repetitions were necessary to obtain one measurement where the hoof was fully measured by the force plate [14]. However, using a set of parallel force plates that were 2.07 m long, on average 3.2 valid steps were obtained in just one repetition [15].

Thorup *et al.* [16] used the 3D force plate system described in Skjøth *et al.* [15] and showed that full vertical force curve symmetry between left and right leg pairs was lower in lame cows compared with non-lame cows. Importantly, they classified non-lame cows as scoring 1, whereas lame cows scored 2-5, thus using a low cut-off. However, their study only included a small number of cows, so the usefullness of full curve symmetry for lameness detection remains to be confirmed by larger studies.

Pastell *et al.* [17] introduced the EmFit, a mat made of electromechanical film which can detect only dynamical forces, into lameness research. Their preliminary test showed that such systems have potential to separate lame from non-lame cows based on the differences in force-time behavior: lame cows used a lower step force and longer stance time with the lame limb compared to the sound limb. A sharper negative peak of the lame limb showed that the painful limb was lifted much faster than the sound limb [17].

#### 2.1.2. Measurement of Standing Cows

In an earlier study, Pastell *et al.* [18] started with automatic measurement of the weight distribution of the cow in a milking robot where the weight of each limb was measured separately using load cells. An algorithm calculated the average weight, the weight variation of each limb and the number and frequency of kicks and steps. Preliminary results on a research farm gave evidence that observed limb and foot disorders could be detected [18]. Pastell and Kujala [19] built a model using a probabilistic neural network. Validation of this model showed 96.2% correct classification of cows as being non-lame or lame, and 100% (Se) of the lameness cases were identified with a specificity of 57.5% (AUC = 0.86). The model could be adjusted to higher specificity at the cost of sensitivity. Of all measurements, only 1.1% were falsely classified as being lame. Pastell and Madsen [20] used CUSUM charts during a long term follow-up study using the cow’s own historical data to detect lameness. They suggested adjusting the specific CUSUM-chart values in order to have a specific level of detection rate and false alarms depending on the use of the system (e.g., on farm for lameness detection, or early lameness detection, or in a veterinary practice for diagnosis). This system was reported to identify lame cows with specific lesions (sole ulcers and white line disease) more quickly than visual locomotion scoring, while joint problems were more easily detected based on the locomotion scores [21]. For the detection of mildly lame cows, repeated measurements on individual cows were suggested to detect changes that occur over time.

Neveux *et al.* [22] used a platform outside the automatic milking system containing four recording units with two load cells each to measure the weight distribution of cows while standing on different surfaces (rubber and concrete). An adjusted set-up was later used by Chapinal *et al.* [23,24] and Pastell *et al.* [25] to measure lameness and hoof lesions. The asymmetry in weight bearing between left and right limbs (Leg Weight Ratio or LWR) was found to be a sensitive measure for detecting severely lame cows (AUC = 0.87). Pastell *et al.* [25] suggested that a cow may suffer pain when walking, which is not as obvious when the cow is standing still. This may be because of several undiagnosed causes behind functional lameness, such as thickened joint capsules, nerve or ligament injury, and muscle problems not affecting standing. They also found that standing LWR measurements were highly correlated with locomotion score for cows with sole ulcers (R² = 0.79), and that some sole ulcers seemed to cause changes in LWR while standing but did not result in visible lameness during walking. On the other hand, LWR was less successful at identifying cows with only haemorrhages, and was unable to discriminate between cows with no hoof lesions and cows with digital dermatitis.

Chapinal and Tucker [26] used a similar system to validate automatically measured steps, while cows stand on the platform. Compared to counting steps on video footage, this system gave a high sensitivity and specificity (>96%) in counting steps.

Using a pressure distribution plate (RsScan), van der Tol *et al.* [27] measured the pressure distribution under one hoof at a time in standing cows. van der Tol *et al.* [28] used this set-up with an addition of a force plate to measure GRF and pressure distributions while walking. However, no tests with lame and non-lame cows were performed with this system.

### 2.2. Pressure-Sensitive Position Mat

**Figure 1:**
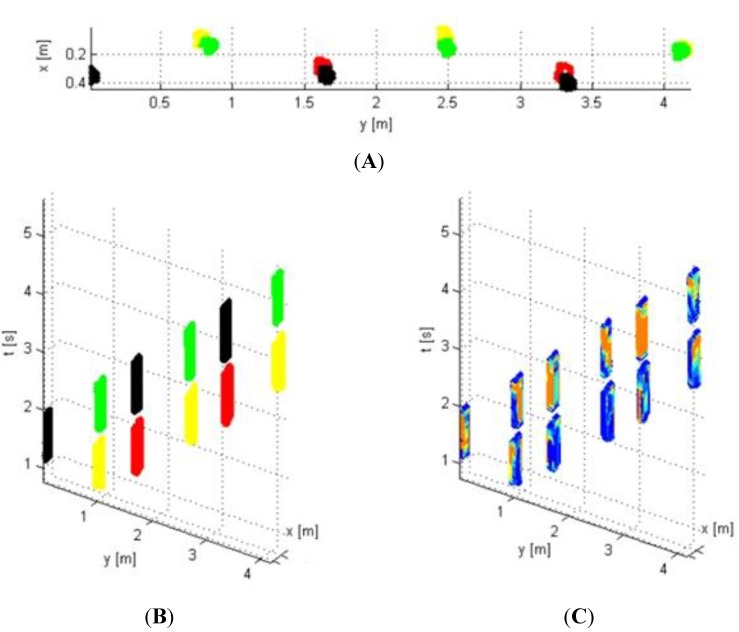
Illustration of the data acquired by the Gaitwise system after data conditioning and identification of each detected imprint. (**A**) Locations of each foot imprint on the measurement zone in x (transverse) and y (longitudinal) coordinates. Each foot was measured three times (Red = right front; black = right hind; yellow = left front; green = left hind). (**B**) The same data are represented with a time axis to show the duration of each imprint. (**C**) Colours on the imprint represent mean pressure from each sensor during each footfall; the relative scale is from grey (lowest relative pressure) through cyan, yellow, magenta and red to blue (highest relative pressure) [78].

Maertens *et al.* [29] developed the Gaitwise system. It is a pressure-sensitive position mat which provides spatio-temporal and relative force information of two complete gait cycles of cows walking over the measurement zone (Figure 1). The Gaitwise system automatically measures the gait of the cows after milking (twice a day) without human interference. Gait variables are available a few seconds after the cow has passed the measurement zone. The raw data of this sensor is transformed to 20 basic variables that describe the general gait of cows walking over the measurement zone together with an additional 10 more specific gait variables that are closely related to gait characteristics used in locomotion scoring systems: “stride length”, “stride time”, “stance time”, “step overlap”, “abduction” and variables covering asymmetry between left and right limbs: “asymmetry in step width”, “asymmetry in step length”, “asymmetry in step time”, “asymmetry in stance time” and “asymmetry in relative force”. A study by Van Nuffel *et al.* [30] revealed that variables of speed and asymmetry measured by the Gaitwise system were closely related to locomotion scores given by observers based on videos of cows walking over the Gaitwise system and matched the Gaitwise variables. The first detection model based on these specific variables could classify 84% of the cows correctly, with a sensitivity of 85%, 76% and 90% and a specificity of 86%, 89% and 100% for the detection of non-lame, mildly lame and severely lame cows respectively [29]. In a follow-up study, variables of gait inconsistency were tested using two case-control studies, revealing the potential of these new variables for the detection of mildly lame cows [31]. Indeed, detection of the mildly lame cows in the model based on the variables of inconsistency improved to a sensitivity of 88% with a specificity of 87% [32]. Moreover, the results of the case control studies suggested that the problem of locating the cause of lameness might be derived from the variables that change significantly [31]. A test-retest study showed that measurements with the Gaitwise system were highly repeatable within cows [31].

### 2.3. Vision Techniques

Flower *et al.* [33] were the first to use vision techniques with body markers to measure temporal and spatial gait characteristics in cows related to lameness. Song *et al.* [34] used video images of walking cows without body markers to automatically measure step overlap as a relevant gait characteristic for lameness detection. Pluk *et al.* [35] wrote an image-based algorithm to calculate the step overlap and compared the output of this algorithm to the locomotion score given by a trained observer. The automatically measured step overlap was found to be highly correlated with manual locomotion scoring (R^2^ = 0.81). In an initial experiment on 15 cows, the measured step overlap seemed to be significant for the distinction between non-lame and mildly lame cows. However, in a second experiment using a simplified scoring system on 104 cows, this distinction was only seen between non-lame and severely lame cows for the minimal step overlap and between mildly and severely lame cows for the maximal step overlap. When combining a camera system with the Gaitwise system, an algorithm to automatically calculate touch and release angles and range of motion was developed [36]. A decreased range of motion and increased release angles of the front legs could detect changes in locomotion scores in a large percentage of the cows (76%).

Another promising variable for lameness detection is the back arch. Poursaberi *et al.* [37] automatically calculated the back arch of each cow during walking by fitting a circle through three selected points on the side view contour line of the back spine. The average inverse radius of four frames displaying the hind feet in contact with the ground (two frames for each hind foot) was calculated for each cow. Based on this curvature value, a score representing the lameness status was given to the individual cow. The results of the algorithm were again compared to locomotion scores resulting in a correct classification of more than 96%. This idea was further elaborated and a Body Movement Pattern was used to describe the movement of the back and head of the cows using a side view [38]. An algorithm based on this Body Movement Pattern was tested under farm conditions by Viazzi *et al.* [39], who reported a correct classification of 81% and 91% using both thresholds, at the population level and at the individual level, respectively.

The algorithm proposed by Viazzi *et al.* [39] was not fully automatic because it still required two manual procedures to select the frames in the video where the hind hooves bear weight and to segment the back contour line from the images. The follow-up study by Van Hertem *et al.* [40] showed that image segmentation in these 2D images was problematic in real farm conditions. Several challenges of the system were summarized in the work of Poursaberi *et al.* [41], Van Hertem *et al.* [40] and Viazzi *et al.* [42]; (1) lack of space on farms to install a side-view camera; (2) changing lighting conditions causing noise and shadows in the images that impede extraction of the back posture and (3) continuous background changes that interfere with cow segmentation from the images. Also, the measurements of Poursaberi *et al.* [37] could not be automated, because the frames had to be manually examined to select the moment in which the cow placed the hind limb on the ground in order to select the following frames to be processed automatically [42].

To overcome the difficulties with back posture analysis based on side-view images from 2D cameras, Van Hertem *et al.* [43] and Viazzi *et al.* [42] tested the use of 3D cameras in top view. The accuracy of the application of both algorithms for detection of lame cows was comparable: 91% and 90% for the 2D and 3D camera systems, respectively [42]. In the study by Van Hertem *et al.* [43], the correct classification rate based on the 5-point scale, a tolerant scale (where one unit difference in locomotion score between the model and reference was accepted) and a binary scale for visual locomotion scoring (non-lame = locomotion score 1 and 2; lame = locomotion score 3 to 5) were 60.2%, 90.9% and 81.2%, respectively. In this study, only cows that were scored within a one-unit score range in four consecutive measurements were used in order to reduce the subjectivity of the observer and to increase reliability of the locomotion scores. Viazzi *et al.* [42] reported that using the 3D camera top view overcomes most of the limitations of the 2D camera approach: (1) top view cameras are easier to install in the barn because no additional space at the side is needed to get good images of the cows; (2) the depth information of the 3D camera made changing backgrounds and shadows less intrusive and enhanced the automatic segmentation of different cows. Limitations of the set-up with the 3D camera were allocated to the high sensitivity of the camera to natural light, small field of view and the lack of information of other gait variables with this approach [42]. The use of 3D cameras allowed fully automatic video recording and processing. However, manual identification of the cows was still needed [43].

Driven by the positive results of infrared thermography for lameness detection in horses [44], this technique was tested to detect hoof lesions in cattle [45]. Both Alsaaod and Büscher [46] and Stokes *et al.* [47] used infrared thermography to investigate the possible role of this technique in detecting the preferred time for trimming or treatment intervention in preventing the occurrence of severe hoof lesions. However, none of these researchers used this technique to divide cows with different degrees of locomotion scores.

### 2.4. Measuring Gait and/or Activity Using Accelerometers

Other studies use accelerometers to measure the activity or gait features of cows and their relation to lameness. In a study by Mazrier *et al.* [48] the reduction in activity (*i.e.*, average steps / hours) for lame cows ranged from 9 to 68% and almost half of the lame cows showed a reduction of more than 5% during the 7 to 10 days prior to clinical signs. In 92% of the lameness cases, the decrease in activity was above 15%. As such devices are currently used for oestrus detection in cattle, Steensels *et al.* [49] also suggested using these devices already present on many farms as a starting point for future research to identify lameness problems. Pastell *et al.* [50] used a custom-made wireless 3D accelerometer system to measure temporal gait characteristics on all 4 limbs of the cows. Differences in symmetry variance and forward acceleration were observed between lame and non-lame cows.

Chapinal *et al.* [51] used five 3D accelerometers on cows, one on each limb and one fastened around the torso. A single device attached to one of the legs appeared to be sufficient to measure the walking speed of cows, which was associated with locomotion scores. In another study accelerometers were mounted on a hind leg on 348 cows in 401 lactations on four commercial farms [52]. On these cows, 959 locomotion scores were assessed by trained technicians, and the activity level differed already between cows of locomotion score 1 and score 2 (on a scale from 1 to 5), thus already detecting the early signs of lameness [52]. Using a sensor attached to one of the front legs of the cows to measure activity and lying behavior, Alsaaod *et al.* [53] were able to predict lameness in cows with an accuracy of 76% based on deviations from normal behavior.

### 2.5. Measuring Lying Time Using Accelerometers

Almost a decade ago, Munksgaard *et al.* [54] suggested the use of sensors that measure acceleration either in 1 or in 3 dimensions to automatically monitor activity or the standing and lying behavior of cows. They found excellent accuracies between the sensor data attached to the legs of the cows and observations for lying and standing (0.99), activity (0.89) and for number of steps (0.84). Since then, a vast number of studies have used accelerometers to measure dairy cow activity and behavior. The results of Munksgaard *et al.* [54] were confirmed by a validation study of O’Driscoll *et al.* [55], where data loggers (1D or 3D accelerometers) attached to the legs of the cows measured activity, lying and standing behaviors in cows with a concordance of 96.3%, suggesting that such sensors are a good alternative to direct behavioral observations.

Similar sensors are used to investigate the link between lying behavior of cows and lameness. The results of Yunta *et al.* [56] suggest that monitoring lying behavior around feeding time could help farmers in finding lame animals, as lame cows stand up later and lie down earlier after fresh feed is delivered compared to non-lame cows. Blackie *et al.* [2] and Calderon and Cook [57] were able to link lameness to different lying time durations and number of lying bouts both before and after calving when comparing non-lame and lame cows.

### 2.6. Combining Already Available Sensor Data

Van Hertem *et al.* [40] used sensors already present on farms to detect clinically lame animals with a sensitivity of 89%, a specificity of 85% and a correct classification rate of 86% using the seven input variables best correlated with lameness, *i.e*., the daily milk yield 4 days before diagnosis, the slope coefficient of the daily milk yield 4 days before diagnosis, the night time to day time neck activity ratio 6 days before diagnosis, the milk yield week difference ratio 4 days before diagnosis, the milk yield week difference 4 days before diagnosis, the neck activity level during the daytime 7 days before diagnosis, and the ruminating time during night time 6 days before diagnosis. Similarly, Kamphuis *et al.* [58] used data from sensors already available on farm such as live weight, activity and milk characteristics. A comparison of those data for a time period of 14 days before a lameness event with control data of non-lame cows resulted in live weight, activity and milking order being selected to have the highest potential for identifying the onset of lameness. Detection models that combine these three variables reached a specificity of about 80%, but the detection performance remained low (50%).

Activity data in terms of lying behavior, combined with milk yield and feeding data in the form of concentrate left-overs in the milking robot, were used to detect lameness by de Mol *et al.* [59] with an overall sensitivity of 86% and specificity of 89%. Liberati and Zappavigna [60] combined measurements of milk production, milk flow and animal activity for detection of abnormal cow health. However, their models were not accurate enough (sensitivity < 56%) for detecting lameness problems in cows. Garcia *et al.* [61] combined 320 variables obtained from a milking robot and neck-based activity meters. Eighty-eight cows were repeatedly locomotion scored on a scale from 1 to 4, then dichotomized into non-lame (score 1) and clinically lame (scores 3 and 4), thus omitting score 2 from analysis. A model for first parity cows and multiparous cows were built, which achieved sensitivity and specificity values around 80%. At the optimum values in the receiver operating characteristic curve, the false-positive rate was 28% in the parity 1 model, whereas in the parity 2 model it was 16%, which makes it more suitable for practical application; the model error rates were 23 and 19%, respectively.

## 3. Practical Considerations in the Development of Lameness Detection Systems

**Discrepancy in the experimental set-ups during validation.** As this review focuses on the development of systems to help farmers in their daily routine, the sensors and technologies summarized were limited to measurements of variables related to ‘visual’ characteristics of lameness in cows that are used in daily practice (e.g., changed locomotion, changed behavior) whether or not combined with information on feeding/drinking behavior, milking process and others (See Table 5). In order for such characteristics to be successfully used in a lameness detection system, the gathered data should be validated by using locomotion scores as a ‘gold standard’ or reference. Locomotion scoring in scientific studies should be performed by a trained observer as most farmers underestimate lameness [62,63]. The practical set-ups and methods of those studies that used locomotion scores as reference to test their measured variable are summarized in Table 1 for studies using load cells, in Table 2 for a pressure sensitive position mat; in Table 3 for computer vision, in Table 4 for accelerometers, and in Table 5 for the combination of already available sensor data. In most of these studies, locomotion scores were obtained by trained observers. However, especially the larger studies combining already available sensor data selected their lame cows based on the observation of the herdsman, not necessarily confirmed by a veterinarian. This approach increases the risk of missing mildly lame cows or even considering severely lame cows in the non-lame group.

More importantly, the **approach in using the locomotion scores as a reference** differs considerably between the different studies. This makes it difficult to compare the different lameness indicators and techniques. Very often, the locomotion of the cows was initially scored based on a 5-point scale ranging from 1 (non-lame) to 5 (severely lame) using different scoring systems. Next, different cut-off values to differentiate between non-lame and lame groups of cows were used (last column in Table 1 to Table 5). Cows with locomotion score 2 (mildly lame) are mostly regarded as ‘non-lame’ and occasionally even cows with locomotion score 3 were considered as ‘non-lame’. As detecting the early stages of lameness could be one of the main advantages of an automated system, such threshold-setting should be carefully considered. For that reason, the sensitivity and specificity of the different systems should also be compared carefully.

In addition, there is large variation in **the number of cows tested** and in the practical set-ups used in the different studies. As discussed in the first part of this review on visual lameness assessment, automated measurements could and should also be done without the interference or obvious presence of an observer. Ideally, measurements are performed during the normal daily routine of the cows (e.g., the balances in the floor of the milking robot). Based on the summary in Table 1 to Table 5, however, more than half of the studies (63%) were performed in experimental set-ups, disturbing the normal daily routine of the cow.

**Table 1:** Summary of experimental set-ups and use of lameness references in studies using load cells.

	*Experimental set-up*						*Lameness reference*		
Source	Sensor type	Number of cows	Normal routine	N° of variables per variable type	Automated measurements	In real- time	Person performing scoring	Number classes used	Cut-off levels for lameness
Rajkondawar *et al.* [8]	2 floor plates with 4 load cells	23	no	weight variables (6)	no	no	herdsman and veterinarian	1 to 5	
Rajkondawar *et al.* [9]	2 floor plates with 4 load cells	31	no	weight variables (5)	yes	yes	observer	1 to 5	classes 1 - 2 - 3
Pastell and Kujala [19]	4 balance system on floor in milking robot	73	yes	weight variables (26)	yes	yes	observer	1 to 5	non-lame: 1 and 2 + no claw lesions lame: 3 and more
Chapinal *et al.* [23]	speed	66	no	speed	no	no	observer	1 to 5	non-lame: 1 - 2 lame: 3 - 4- 5
	weighing platform with 4 balances			weight variables (12)	no				
	Pedometer			lying behaviour (2)	yes				
Chapinal *et al.* [24]	speed	57	no	speed	no	no	observer	1 to 5	non-lame: 1 - 2 lame: 3 - 4- 5
	weighing platform with 4 balances			weight variables (12)	no				
	Pedometer			lying behaviour (2)	yes				
Pastell *et al.* [25]	weighing platform with 4 balances	55	no	weight variables (16)	no	no	observer	1 to 5	non-lame: 1 - 2 lame: 3 - 4- 5
Liu *et al.* [12]	StepMetrix	346	no	weight variables (5) and symmetry variables	yes	yes	observer	1 to 5	non-lame: 1 -2 (3) lame: (3) - 4 - 5
Chapinal and Tucker [26]	weighing platform with 4 balances	57	no	steps (validate by camera observations + frequency of steps + weight shifting	no	no	observer	1 to 5	non-lame: 1 - 2 - 3 lame: 4 - 5
Thorup *et al.* [16]	2 force plates with 4 load cells	9	no	Full curve symmetry in 3 dimensions	no	no	observer	1 to 5	Non-lame: 1 lame: 2 - 5

Normal Routine: cows walk in normal routine or were guided in an experimental set up: Automated: measurements were automated without presence of an operator; real-time: results of variables are available in real-time.

**Table 2:** Summary of experimental set-ups and use of lameness references in studies using position sensors.

	*Experimental set-up*						*Lameness reference*		
Source	Sensor type	Number of cows	Normal routine	N° of variables per variable type	Automated measurements	In real- time	Person performing scoring	Number classes used	Cut-off levels for lameness
Maertens *et al.* [29]	Pressure sensitive mat	159	yes	Basic gait variables (20) Specific gait variables (10)	yes	yes	observer	1 to 3	non-lame: 1 mildly lame: 2 severely lame: 3
Van Nuffel *et al.* [31]	Pressure sensitive mat	40	yes	Basic gait variables (20) Gait inconsistency variables (20)	yes	yes	observer	1 to 3	non-lame: 1 mildly lame: 2 severely lame: 3
Van Nuffel *et al.* [32]	Pressure sensitive mat	36	yes	Basic gait variables (20) Gait inconsistency variables (20)	yes	yes	observer	1 to 3	non-lame: 1 mildly lame: 2 severely lame: 3

Normal Routine: cows walk in normal routine or were guided in an experimental set up: Automated: measurements were automated without presence of an operator; real-time: results of variables are available in real-time.

**Table 3:** Summary of experimental set-ups and use of lameness references in studies using computer vision.

	*Experimental set-up*						*Lameness reference*		
Source	Sensor type	*Number of cows*	*Normal routine*	N° of variables per variable type	Automated measurements	In real- time	Person performing scoring	Number classes used	Cut-off levels for lameness
Song *et al.* [34]	digital camera	15	no	1 (trackway overlap)	no	no	observer	1 to 5	all classes separate
Pluk *et al.* [35]	digital camera	15/66	no/yes	1 (trackway overlap)	no/yes	no/no	observer	1 to 5	all classes separate
Pluk *et al.* [36]	digital camera combined with Gaitwise-system	70/75	yes/yes	3 (touch angle, release angle, range of motion in the fetlock joint)	no/yes	no/no	observer	1 to 3	all classes separate
Poursaberi *et al.* [37]	digital camera	28/66	yes/yes	1 (back posture)	no/no	no/no	observer	1 to 3	all classes separate
Blackie *et al.* [64]	leg markers, digital camera	56	no	7 (stride length front and hind, tracking distance, hock flexion, max fetlock height, height of spine, head position)	no	no	observer	1 to 5	all classes separate (no 4 and 5 used)
Viazzi *et al.* [39]	digital camera	98	yes	1 (body movement pattern)	no	no	observer	1 to 5	non-lame: 1 + 2 lame: 3 severely lame: 4 + 5
Van Hertem *et al.* [43]	3D-digital camera	186	yes	1 (back posture measurement)	yes	no	observer	1 to 5	- all classes separate; - all classes separate with 1 level tolerance; - non-lame: 1 + 2 lame: 3 – 5
Viazzi *et al.* [40]	digital camera and 3D-digital camera	273	yes	1 back posture in 2D 1 back posture in 3D	no/yes	no/no	observer	1 to 5	non-lame: 1 + 2 lame: 3 - 5

Normal Routine: cows walk in normal routine or were guided in an experimental set up: Automated: measurements were automated without presence of an operator; real-time: results of variables are available in real-time.

**Table 4:** Summary of experimental set-ups and use of lameness references in studies using accelerometers.

	*Experimental set-up*						*Lameness reference*		
Source	Sensor type	Number of cows	Normal routine	N° of variables per variable type	Automated measurements	In real-time	Person performing scoring	Number classes used	Cut-off levels for lameness
Mazrier *et al.* [48]	pedometer on hind leg	400	yes	activity (1)	yes	no	herdsman		
Pastell *et al.* [50]	pedometers both hind legs	6 non-lame 6 severely lame	no	activity (6)	no	no	observer	1 to 5	non-lame: 1 + 2 lame: 4
Ito *et al.* [65]	pedometers	1319	yes	4 (lying behaviour)	yes	no	observer	1 to 5	non-lame: 1 + 2 lame: 3 severely lame: 4 + (5)
Blackie *et al.* [2]	markers and video images pedometers	25	no yes	gait variables (7) lying behaviour (6) activity	no yes yes	no no no	observer	1 to 5	all (no 4 and 5 present)
Calderon and Cook [57]	pedometer on hind leg	57	yes	3 (lying behaviour)	yes	no	observer	1 to 4	non-lame: 1 lame: 2 severely lame: 3
Chapinal *et al.* [51]	accelerometers (all 4 legs + around torso)	12/24	no	Speed accelation variables (2)	no	no	observer	1-5 + VAS	all
Alsaaod *et al.* [53]	pedometers on front leg	30	no	activity (1) 4 (lying behaviour)	yes	no	observer	1 to 5	non-lame: 1 + 2 lame: 3 severely lame: 4
Yunta *et al.* [56]	pedometers	250	yes	4 (lying behaviour)	yes	no	observer	1 to 5	non-lame: 1 lame: 3 + 4
Navarro *et al.* [66]	pedometers	400	yes	standing and lying time	yes	no	observer	1 to 5	non-lame: 1
Thorup *et al.* [52]	Accelerometer on 1 hind leg	348	yes	13	no	no	observer	1 to 5	non-lame: 1 lame: 2 - 5

Normal routine: cows walk in normal routine or were guided in an experimental set up: Automated: measurements were automated without presence of an operator; real-time: results of variables are available in real-time.

**Table 5:** Summary of experimental set-ups and use of lameness references in studies using available sensor data on farm.

	*Experimental set-up*						*Lameness reference*		
Source	Sensor type	Number of cows	Normal routine	N° of variables per variable type	Automated measurements	In real-time	Person performing scoring	Number classes used	Cut-off levels for lameness
De Mol *et al.* [59]	activity data (7)	100	yes	activity data (7)	yes	no	herdsmen	1 to 5	non-lame: 1 lame: 3 - 4 - 5 (score 2 excluded)
Kramer *et al.* [67]	milkmeters	81	yes	milk yield	yes	Yes	herdsman	/	Logbook lameness events
	feeding and drinking behaviour			feeding behavior (4)		No			
	activity meters (neck)			activity		No			
	farm health records			info preliminary diseases		No			
Miekley *et al.* [68]	pedometers	653	yes	activity data (1)	yes	no	herdsmen and veterinarian		Logbook lameness events
	milking data			milking data (2)					
	feeding data			feeding data (3)					
Miekley *et al.* [69]	pedometer activity	315	yes	activity data (1)	yes	no	herdsmen and veterinarian		Logbook lameness events
	feeding patterns			feeding data (3)					
	milking data (1)			milking data (1)					
	feeding data (1)			feeding data (1)					
Kamphuis *et al.* [58]	weight scales	318 lame and 3180 non-lame	yes	weight scales (1)	yes	yes	(trained) herdsmen	1 to 5	Logbook lameness events
	pedometers			pedometers (1)		no			
	milk meters			milk meters (4)		yes			
Van Hertem *et al.* [40]	neck activity	44 lame and 74 non-lame	yes	neck activity (1)	yes	no	herdsmen		Logbook lameness events
	ruminating time			ruminating time (1)		no			
	milking data			milking datad (5)		yes			
Garcia *et al.* [61]	milking data neck activity	88	yes	milking data (320) activity index	no	no	observer	1 to 4	non-lame: 1 lame: 3 + 4 (score 2 excluded)
Norring *et al.* [70]	automatic feeders milking data weight scales	50	yes	Feeding behavior (4) milk yield and milking frequency body weight	yes	no	observer	1 to 5	non-lame: 1 + 2 mildly lame: 3 severely lame: 4 + 5

Normal routine: cows walk in normal routine or were guided in an experimental set up; Automated: measurements were automated without presence of an operator; Real-time: results of variables are available in real-time.

Finally, if data and lameness classification results were to be available in real-time, cows in need of treatment could be separated from the herd automatically. Obviously, such a procedure would require barn equipment where the cows can be identified and then guided to a separate area (e.g., after milking). In three out of four studies, the variables were calculated afterwards, and calculations were not always performed fully automated. This means some additional integration work would be needed to obtain a real-time automatic lameness detection system.

**Need for automated and continuous measurements?** Automated measurements can gather data continuously, such that cows can be monitored on a daily basis. In addition, their major advantage is the lack of need for herding the cows. As cows have a stoic nature, guiding them can bias the measurements, because they will try to hide their weakness and pain compared to measurements during normal routine without the presence of a human or predator [71]. Ideally, measurements should therefore be fully automated, so no interference of an operator is necessary. Based on the research in Table 1 to Table 5, only 63% of the studies measured cows during normal farming routine without additional guiding. In studies using off-the-shelf accelerometers or commercially available sensors, four out of five measurements were performed without guiding the cows, and in about 82% of these studies, experiments were conducted automatically.

**Need for ‘early’ detection?** Throughout the past 20 years of research on dairy cow lameness and automatic lameness detection systems, scientists have claimed that early detection of lameness signs is beneficial in treating affected animals before the problem becomes too severe. In doing so, long lasting and costly treatments, production losses and reduced welfare long term can be avoided. From a theoretical point of view this might indeed be true, but implementing the concept of “early lameness detection” in practice poses reasonable questions: What is early detection? When is it needed or when does a farmer perceive it as an added value? Nowadays, the biggest challenge for any lameness detection system is the detection of early onset. The definition of ‘early detection’, however, is not so clear, as ‘early’ can be considered in several ways.

A first approach for early detection is ‘detection before the visual clinical signs of lameness are present’. Even though this is the most logical interpretation, this might not be relevant for a lameness detection tool on commercial farms. It can be expected that farmers will not start treatment before obvious signs of lameness are present. According to a study of Alawneh *et al.* [72], over 65% of the mildly lame cows and more than 40% of the severely lame cows were treated more than three weeks after detection. One of the reasons why farmers postpone treatment or pay less attention to lame cows in general is the work load at peak times, such us sowing time in spring or harvesting time in late summer or autumn. The farmers’ priorities simply shift to other tasks [73]. As several hoof lesions take some time to develop, it might also be difficult for hoof trimmers or veterinarians to define the lesion causing the lameness and give accurate treatment at such early stages. Identification of problems in hoof shape, on the other hand, might provide information for the optimal timing to apply preventive hoof trimming. However, early detection of lameness is a prerequisite for effective treatment, which again may prevent the lameness from becoming chronic [74,75]). Therefore, another way to approach ‘early’ detection might be ‘before the lame cow would be noticed by the farmer’ or even ‘at the same time’. Hence, automated lameness detection would more or less be an automation of the daily visual locomotion scoring. Such an approach to early detection seems most feasible for any of the detection systems for lameness and still fits the scope of supporting the farmer in monitoring the individual cows.

**Need for custom-made detection systems?** Early detection of lameness should be used only if it is relevant to the farmer, which suggests that there may be a need for custom-made detection systems. For a farmer with low herd lameness prevalence and a good general lameness management, early detection of new cases of lameness or mildly lame cows might create an added value. However, such farmers are rare. Most farmers hugely underestimate not only the prevalence of the lame cows in their herds [62,63], but also the severity of the lameness cases [63,76]. The majority of farmers prefer a detection system which only creates alarms for the severe cases (specificity of >99%), as shown for automated detection of mastitis [77]. Severe cases can be divided into new cases or chronic cases (*i.e.*, those that cause recurring alarms). In the next step, after prevention and treatment programs have successfully resulted in a lower lameness prevalence at the herd, the threshold settings of the detection system could be changed to also detect the mildly lame cows to further decrease the lameness prevalence to a lower level. In general, it is very important to convince the farmer to trust the lameness detection system. Many farmers may be reluctant to rely on the judgement of an automatic system rather than their own. The farmer’s suspicion might be even worse for early lameness detection, especially if he or she cannot diagnose a treatable problem. In general, a lameness detection system should provide the ideal balance between detecting almost every lame cow (high sensitivity) and having as few false detections as possible (high specificity). Farmers might be more reluctant about a high number of false alerts compared to a missed lame cow, as false alerts create unnecessary labor and time to check the cows on the alert list. However, research on the expectations and use of farmers for a lameness detection system might provide more information on the requested sensitivity and specificity of lameness detection systems.

**Need for real-time measurements?** If the detection could be performed in real-time, *i.e.*, immediately after the cow was measured by the system, it becomes possible to automatically separate a cow that is identified as lame by the software and needs extra attention from the farmer. Such decisions made on the spot must be done in less than five seconds, requiring technology with high-speed calculations.

Of the measurement approaches shown in Table 1 to Table 5, data were only available in real-time in fewer than one out of four studies. The fact that variables measured with accelerometers were not considered real-time because they were mostly based on averaged values over a period of 24 hours might have caused this low number of real-time measurement systems. However, analysis of accelerometer data can be performed e.g., during milking, thereby allowing real-time analysis. No study on vision techniques analyzed the images directly after the experiment period and in situ. Whether or not measurements of a lameness detection system should be in real-time at this stage of the development process remains unclear. Farmers willing and flexible enough to treat lame cows every day may benefit from real-time measurements. Farmers planning fixed weekly treatment days may not need real-time systems because cows that were identified lame in the past week could be selected and separated on one specific day. One might also suggest that based on the list with attention cows, a farmer should decide which cow should be examined and hence which cows have to be separated from the group after next milking. For the top managers with smaller herds or fewer problems, this approach might indeed create less frustration as such farmers have the skills and knowledge to decide which cow should or should not receive treatment. On the other hand, an automatically separated cow would force the farmer to attend to the separated cow before releasing her back into the barn. This would enhance the chance of that cow getting proper treatment. Systems working in real-time always have the advantage of decisions being made in real-time without being a necessity. Real-time data can also be stored and processed later depending on the application and the farmer’s request. All options remain possible. Whether or not lameness detection will be a real-time solution may also depend on interested companies and what they envision as a commercial application that can be sold to their clients at a reasonable price.

**Available space.** In practice, no free space is available in dairy barns to install any lameness detection system. This might create drawbacks for those sensor technologies that need an alley set-up where measurements are performed (walk-over devices, e.g., the Gaitwise system, StepMetrixTM) or where video can be recorded (vision techniques). Therefore, measurement systems that need less space or that can be included in the existing farm infrastructure, like measurements of weight differences in the milking robot or measurements with accelerometers, might be more feasible in practice. If not, additional space could be incorporated in the plans for new barns. However, creating sufficient space in any existing barn is challenging, especially as cows should preferably pass this measurement zone daily—if possible, after milking—and should be identified simultaneously. This requires a free zone inside the barn after the milking parlor, rotary or milking robot or even at the exit to the pasture for measurements during the grazing season. This drawback however, is especially present in the smaller dairy farms. In larger dairies, which are the kind of farms which would benefit hugely from this technology, installing a lameness detection system might be easier. However, the most important thing is to ensure good cow traffic, especially if a system is installed after the milking parlor or milking robot. Cows blocking the area around the measurement zone will disrupt the measurements and create measurement failures and might even affect the milking routine [32].

## 4. Conclusions

In spite of the amount of research available on measurement of gait and behavioral characteristics that are relevant to lameness detection, no efficient automated lameness detection system is available on the market yet. This review focused on the discrepancy between the experimental set-ups used in the studies, the stage of automation of the measurements, and the practical considerations when implementing a lameness detection system on farm. Most research on lameness detection focuses on the detection of severely lame cows, often ignoring mildly lame cows or considering them non-lame. On the other hand, the practical feasibility of also detecting the mildly lame cases should be investigated on farms. This might result in custom-made lameness detection systems that are adjustable depending on the degree and severity of mildly and severely lame cases on that farm and the preferences of the farmer for specific characteristics of the system. In addition, several sensor technologies take up quite some space and will need to be very cost-efficient in order for farmers to decide to buy and create the needed space for the installation.
